# Orbital rhabdomyosarcoma with skin metastasis: a case report

**DOI:** 10.1186/1756-0500-7-670

**Published:** 2014-09-24

**Authors:** Fadwa Elomrani, Salima Touri, Imane Ouziane, Narjiss Berrada, Saber Boutayeb, Hind Mrabti, Basma Elkhannoussi, Hassan Errihani

**Affiliations:** Department of Medical Oncology, National Institute of Oncology, Rabat, Morocco; Department of Pathology, National Institute of Oncology, Rabat, Morocco

**Keywords:** Rhabdomyosarcoma, Cutaneous metastasis, Immunohistochemistry, Treatment, Chemotherapy, Radiotherapy

## Abstract

**Background:**

Rhabdomyosarcoma is a soft tissue neoplasm that usually arises in the headand neck region and genitourinary tract. Skin metastasis of rhabdomyosarcoma is extremely rare; of thirteen cases reported in the literature, most were children younger than 10 years and only three cases have been reported in adults.

**Case presentation:**

A 20-year-old Moroccan man was admitted with a right orbital tumor. The tumor was excised and histopathology examination confirmed a diagnosis of rhabdomyosarcoma. The patient was treated with chemotherapy, but local recurrence occurred one year later. The patient underwent right orbital exenteration followed by chemotherapy and radiotherapy. After 6 months, the patient developed a cutaneous mass in the right lumbar region, which was resected. Immunohistochemical examination of the tumor showed this to be a cutaneous metastasis of rhabdomyosarcoma. The patient was treated by chemotherapy and there appeared to be no recurrence after 9 months of follow up.

**Conclusions:**

Skin metastasis from rhabdomyosarcoma is extremely rare, particularly in adults. The purpose of presenting this case report is to raise awareness among clinicians— skin biopsy and immunohistochemistry are needed to distinguish this neoplasm from other cutaneous tumors so that appropriate treatment can be initiated.

## Background

Rhabdomyosarcoma (RMS) is the most common soft-tissue sarcoma in childhood and adolescence. Approximately 350 new cases occur each year in the US [1]. The most commonly involved sites at presentation are genitourinary (29%), parameningeal (24%), the extremities (15%), retroperitoneal (13%), orbit (8%), and other head and neck areas (7%) [1]. Orbital (RMS) is the most common primary orbital malignancy in children with approximately 35 new cases per year [2]. The treatment of choice is chemotherapy and radiation as determined by the Intergroup Rhabdomyosarcoma Study [3]. Orbital RMS has a relatively good prognosis, 70% of children and adolescents with RMS are cured [4, 5]. Cutaneous metastasis of RMS is rarely described in the literature; between 1966 and 2014 only 13 cases were reported [6–15]. Among these cases, only three were in adults [6–8]. We report a case of a 20-year-old Moroccan man with orbital rhabdomyosarcoma who developed skin metastasis.

## Case presentation

A previously fit and well 20-year-old Moroccan man presented with a right orbital tumor measuring 2 cm in diameter that had been rapidly increasing in volume. A complete resection of the tumor was made. The diagnosis was made on histological examination, confirming the orbital tumor to be an embryonal rhabdomyosarcoma.

**Figure 1:**
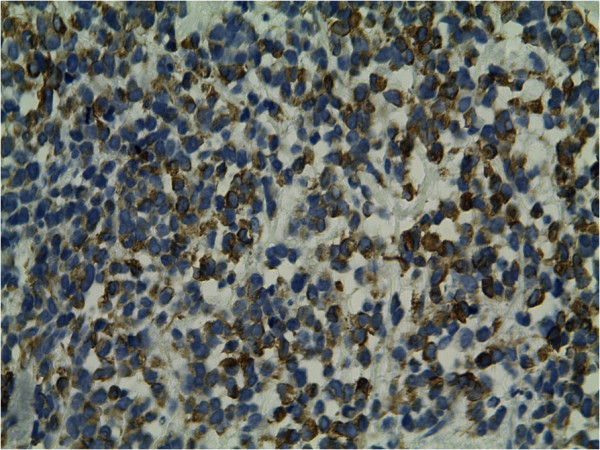
**Subcutaneous metastasis of round tumor cells,** **original magnification X10.**

**Figure 2:**
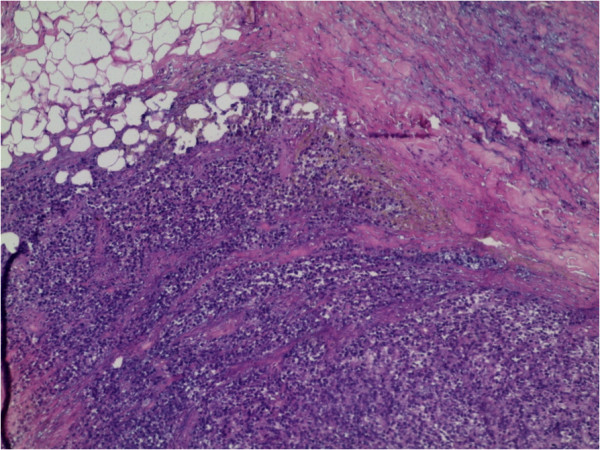
**Groups of round tumor cells with hyperchromatic nuclei,** **original magnification X40.**

The patient received six cycles of adjuvant chemotherapy with alternating vincristine, adriablastine and cyclophosphamide(VAC). One year later, the patient developed a local recurrence. Exenteration was performed,followed by treatment with six cycles of ifosfamide plus etoposide (IE); local radiotherapy was also given at a dose of 45 Gy. However, after 6 months, the patient developed a cutaneous mass in the right lumbar region, which was resected. A computed tomography - scan of the thorax, abdomen and pelvis did not reveal any metastasis. Macroscopically, the tumor size was 4 × 4 × 2.2 cm; the cut surfaces were nodular and fleshy; there was no necrosis and the edges showed an infiltrative pattern. Histologic sections with microscopy showed a proliferation of undifferentiated round cells arranged in sheets with some tapered eosinophilic cytoplasm. High mitotic rate was seen. The tumor cells infiltrating the subcutaneous tissue. Hematoxylin and eosin stained sections revealed loosely arranged round cells with large hyperchromatic nuclei and rare strap cells with eosinophilic cytoplasm (Figures 1 and 2). Immunohistochemical studies were performed using antibodies directed against desmin,myogenin, cytokeratin and leukocyte common antigen(LCA).

**Figure 3:**
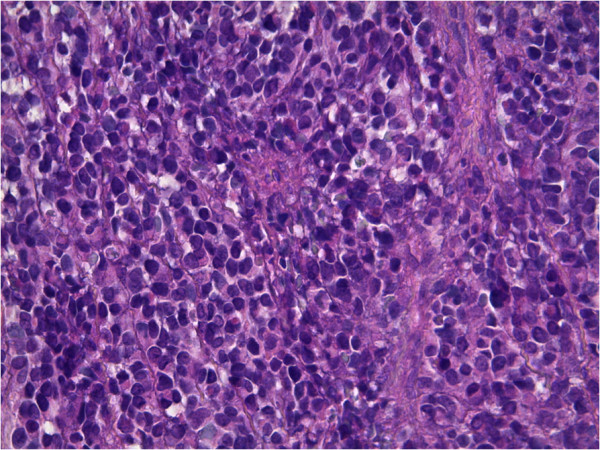
**Immunohitochemistry study reveals strong positivity for desmin.**

**Figure 4:**
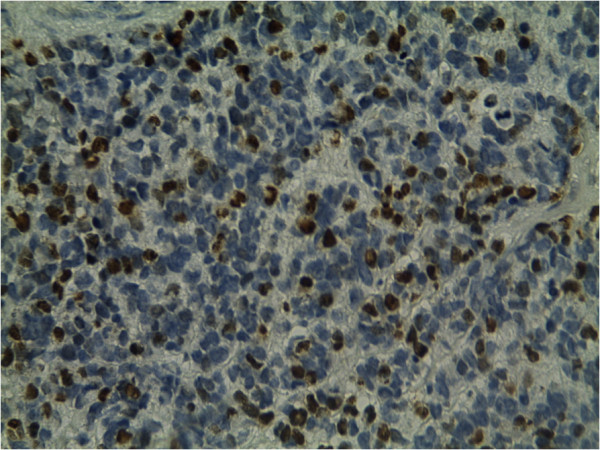
**Immunohitochemistry study reveals strong positivity for myogenin.**

Photomicrographs showing a sheet of undifferentiated round cells. The tumour cells were immunopositive for desmin (Figure 3) and myogenin (Figure 4), and negativity for cytokeratin and LCA. A final diagnosis of cutaneous RMS metastasis was made. The patient then was treated by chemotherapy with 6 cycles of IE. He is still living 9 months after the initial diagnosis of skin metastasis with no recurrence.

## Discussion

RMS is an uncommon neoplasm in the adult population. Metastasis to skin is extremely rare in both children and adults with RMS. Only three adult cases of RMS with skin metastases have been reported in the literature [6–8]. Most of these cases were treated by surgical excision, chemotherapy and radiotherapy, but the prognosis was poor. The 5-year overall survival in patients with distant metastases is 20% to 30% from three published trials [16]. According to the literature, RMS of the skin is often misdiagnosed and has similar clinical features to other more common soft tissue tumors [17].

Most cases of RMS skin metastasis are of the alveolar subtype, which is most often seen in pediatric patients [18]. It is often very difficult to differentiate the skin metastases of RMS from other skin neoplasms that are composed of spindle or round cells, such as lymphoma, melanoma, leiomyosarcoma, neuroblastoma, Merkel cell carcinoma and extra-skeletal Ewing’s sarcoma [19]. The use of immunohistochemistry may provide additional information to aid in differential diagnosis. Positivity for LCA in lymphoma, CD99 immunoreactivity and EWS/Fli1 translocation in extraskeletal Ewing sarcoma. 80% of S100 protein and HMB45 are positive in Melanoma, CD56 strongly positive in neuroblastoma. Most Merkel cell carcinoma are usually positive to cytokeratin 20, neuron-specific enolas, and neurofilament. Leiomyosarcoma are usually positive for desmin and smooth muscle actin [20]. The diagnosis of RMS is made by immunohistochemistry, the presence of cross striations with positivity for MyoD1, myogenin and desmin help to make the diagnosis [18].

Cytogenetic analysis plays an important role in confirming the diagnosis, but only for alveolar RMS because there is no specific cytogenetic or molecular markers for embryonal RMS. Alveolar RMS is associated with a specific translocation, t(2;13)(q37;q14) or its variant t(1;13)(p36;q14) [21].

Treatment for this tumor is multimodal, including surgical excision with or without radiotherapy and chemotherapy. The prognosis of this disease depends on the location and number of metastatic sites, and the treatment received [5]. The 13 cases described in the literature had a median survival of 8 months [6].

## Conclusions

Embryonal RMS with skin metastasis is quite rare and can readily be misdiagnosed. Skin biopsy with immunohistochemistry is necessary to make the diagnosis and to distinguish this malignancy from others cutaneous conditions, so that adequate treatment can be initiated.

## Consent

Written informed consent was obtained from the patient for publication of this Case Report and any accompanying images. A copy of the written consent is available for review by the Editor-in-Chief of this journal.

## Abbreviations

RMS: Rhabdomyosarcoma
VAC: vincristine-adriablastine-cyclophosphamide
IE: ifosfamide-etoposide
LCA: leukocyte common antigen.
